# Durvalumab supplementation for non-small-cell lung cancer: a meta-analysis study

**DOI:** 10.1186/s13019-024-02940-3

**Published:** 2024-07-04

**Authors:** Chengchen Wang, Hongyi Fu, Feng Wang

**Affiliations:** 1 Department of Oncology Radiotherapy Center, Chongqing University Cancer Hospital, Chongqing, 400030 China; 2 Department of Nursing, The First Affiliated Hospital of Chongqing Medical University, Chongqing, 400042 China; 3 Chongqing University Cancer Hospital, No. 181 Hanyu Road, Chongqing, 400030 China

**Keywords:** NSCLC, Durvalumab, Overall survival, Progression-free survival

## Abstract

**Background:**

Durvalumab supplementation may have some potential in improving the efficacy in patients with non-small-cell lung cancer (NSCLC), and this meta-analysis aims to explore the impact of durvalumab supplementation on efficacy for NSCLC.

**Methods:**

PubMed, EMbase, Web of science, EBSCO, and Cochrane library databases were systematically searched, and we included randomized controlled trials (RCTs) assessing the effect of durvalumab supplementation on efficacy in patients with NSCLC. Overall survival and progression-free survival were included for this meta-analysis.

**Results:**

Four RCTs were finally included in the meta-analysis. Overall, compared with control group for NSCLC, durvalumab supplementation showed significantly improved survival rate (odd ratio [OR] = 1.64; 95% confidence interval [CI] = 1.31 to 2.06; *P* < 0.0001), overall survival ( hazard ratio [HR] = 0.73; 95% CI = 0.61 to 0.87; *P* = 0.0003), progression-free survival rate (OR = 2.31; 95% CI = 1.78 to 3.01; *P* < 0.00001) and progression-free survival (HR = 0.71; 95% CI = 0.54 to 0.95; *P* = 0.02), and had the capability to reduce the incidence of grade ≥ 3 adverse events (OR = 0.26; 95% CI = 0.16 to 0.42; *P* < 0.00001).

**Conclusions:**

Durvalumab supplementation is effective to improve the efficacy for NSCLC.

**Supplementary Information:**

The online version contains supplementary material available at 10.1186/s13019-024-02940-3.

## Introduction

Immune checkpoint inhibitors targeting programmed cell death-1 (PD-1) or its ligand programmed cell death ligand-1 (PD-L1) have important potential in treating non-small-cell lung cancer (NSCLC) [1–5]. However, not all patients with NSCLC can obtain immunotherapy in the first- or second-line setting because of many factors including approval/availability of these products at that time in some countries [6]. More therapy options are needed for patients with disease progression.

As one selective, high-affinity, human immuno-globulin G1 monoclonal antibody, durvalumab has shown the promise for the treatment of patients with unresectable, stage III NSCLC by blocking PD-L1 binding to PD-1 and CD80 [7, 8]. Especially, in patients with PD-L1 expression levels, anti-PD-1/PD-L1 monotherapies was reported to significantly improve the efficacy outcomes of lung cancers [9–11].

Several RCTs showed that durvalumab supplementation may have the capability to improve the efficacy for NSCLC, but the results were not well established [6, 8, 12]. Considering these inconsistent effects, we therefore conducted a systematic review and meta-analysis of RCTs to evaluate the effectiveness of durvalumab supplementation versus standard chemotherapy on treatment efficacy for NSCLC.

## Materials and methods

### Study selection and data collection

This meta-analysis of previously studies did not need ethical approval or patient consent. It was conducted according to the Preferred Reporting Items for Systematic Reviews and Meta-analysis statement and Cochrane Handbook for Systematic Reviews of Interventions [13, 14].

We have searched PubMed, EMbase, Web of science, EBSCO, and the Cochrane library up to June 2022, using the search terms “durvalumab” AND “lung cancer” OR “NSCLC”. The inclusion criteria were as follows: (1) study design was RCT; (2) patients were diagnosed with NSCLC; (3) intervention treatments were durvalumab versus standard chemotherapy.

### Assessment for risk of bias

The risk of bias tool was used to assess the quality of individual studies in accordance with the *Cochrane Handbook for Systematic Reviews of Interventions* [15], and the following sources of bias were considered: selection bias, performance bias, attrition bias, detection bias, reporting bias, and other potential sources of bias. The overall risk of bias for each study was evaluated and rated: low, when the risk of bias was low in all key domains; unclear, when the risk of bias was low or unclear in all key domains; and high, when the risk of bias was high in one or more key domains [16]. Two investigators independently searched articles, extracted data, and assessed the quality of included studies. Any discrepancy was solved by consensus.

### Outcome measures

The following information was extracted: first author, publication year, sample size, age, male, tumor histologic subtype (squamous/nonsquamous) and methods of two groups. The primary outcomes were survival rate and overall survival. Secondary outcomes included progression-free survival rate, progression-free survival, adverse events, and grade ≥ 3 adverse events.

### Statistical analysis

A team consisting of three authors did the statistical analyses. Hazard ratio (HR) with 95% confidence interval (CI) was used to assess continuous outcomes and odd ratio (OR) with 95% CI was used to assess dichotomous outcomes. I^2^ statistic was used to assess the heterogeneity, and significant heterogeneity was observed when *I*^*2*^ > 50% [17]. The random-effect model was used regardless of the heterogeneity. We conducted the sensitivity analysis through detecting the influence of a single study on the overall estimate via omitting one study in turn or using the subgroup analysis. *P<* 0.05 indicated statistical significance and Review Manager Version 5.3 was used in all statistical analyses.

## Results

### Literature search, study characteristics and quality assessment

**Fig. 1:**
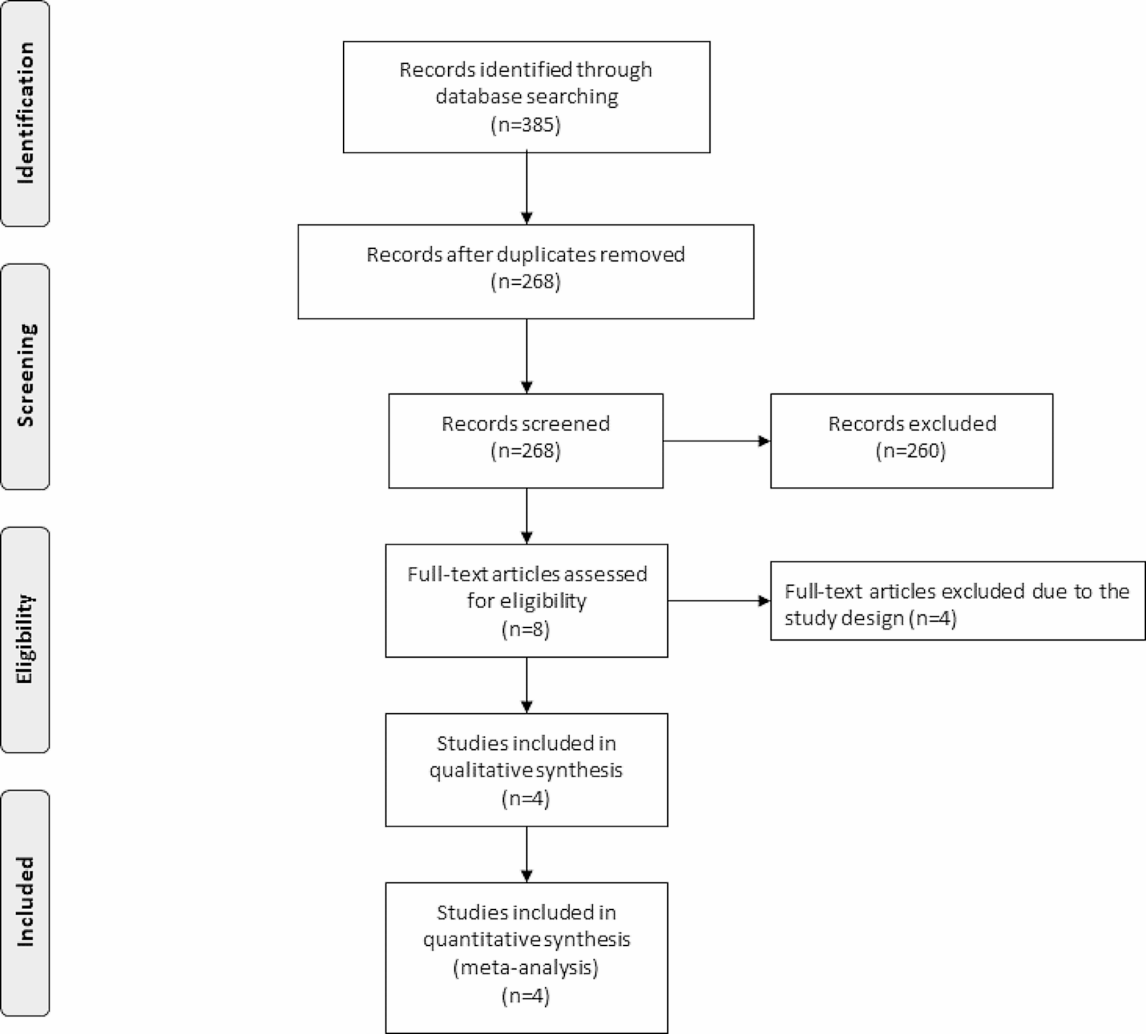
Flow diagram of study searching and selection process

The flow chart for the selection process and detailed identification was presented in Fig. 1. 385 publications were identified through the initial search of databases. Ultimately, four RCTs were included in the meta-analysis [6, 8, 12, 18].

The baseline characteristics of the four eligible RCTs in the meta-analysis were summarized in Table 1. The four studies were published between 2017 and 2020, and total sample size was 1399. There were similar baseline characteristics between durvalumab group and control group. The treatment duration of durvalumab supplement were different in each RCT, ranging from 2 years to 3 years. Two studies reported the same patient sample with different outcomes [8, 18].

**Table 1:** Characteristics of included studies

NO.	Author	Durvalumab group					Control group							
		Number	Age (years)	Male (*n*)	Tumor histologic subtype (squamous/ nonsquamous)	Methods	Number	Age (years)	Male (*n*)	Tumor histologic subtype (squamous/ nonsquamous)	Methods	Stages	Previous treatment course	Median follow-up time
1	Rizvi 2020	163	64.0 (32–84), median (range)	113	52/111	durvalumab (20 mg/kg every 4 weeks) for 4 months	162	64.5 (35–85)	106	52/110	standard chemotherapy	stage IV	no previous systemic therapy	30 months
2	Planchard 2020 (A)	62	63.5 (35–79), median (range)	42	16/46	durvalumab 10 mg/kg every 2 weeks for up to 12 months	64	62.0 (41–81)	48	16/48	standard chemotherapy	stage IIIB/IV	platinum-doublet regimen and one or more additional systemic regimens	30 months
	Planchard 2020 (B)	117	63.0 (19–83)	73	29/88		118	65.0 (42–83)	81	28/90				
3	Antonia 2018	476	64(31–84), median (range)	334	224/252	durvalumab 10 mg/kg intravenously every 2 weeks as consolidation therapy for up to 12 months	237	64 (23–90)	166	125/107	standard chemotherapy	stage III	at least two cycles of platinum-based chemotherapy and definitive radiation therapy	2 years
4	Antonia 2017	476	64(31–84), median (range)	334	224/252	durvalumab 10 mg/kg intravenously every 2 weeks as consolidation therapy for up to 12 months	237	64 (23–90)	166	125/107	standard chemotherapy	stage III	two or more cycles of platinum-based chemoradiotherapy	2 years

**Fig. 2:**
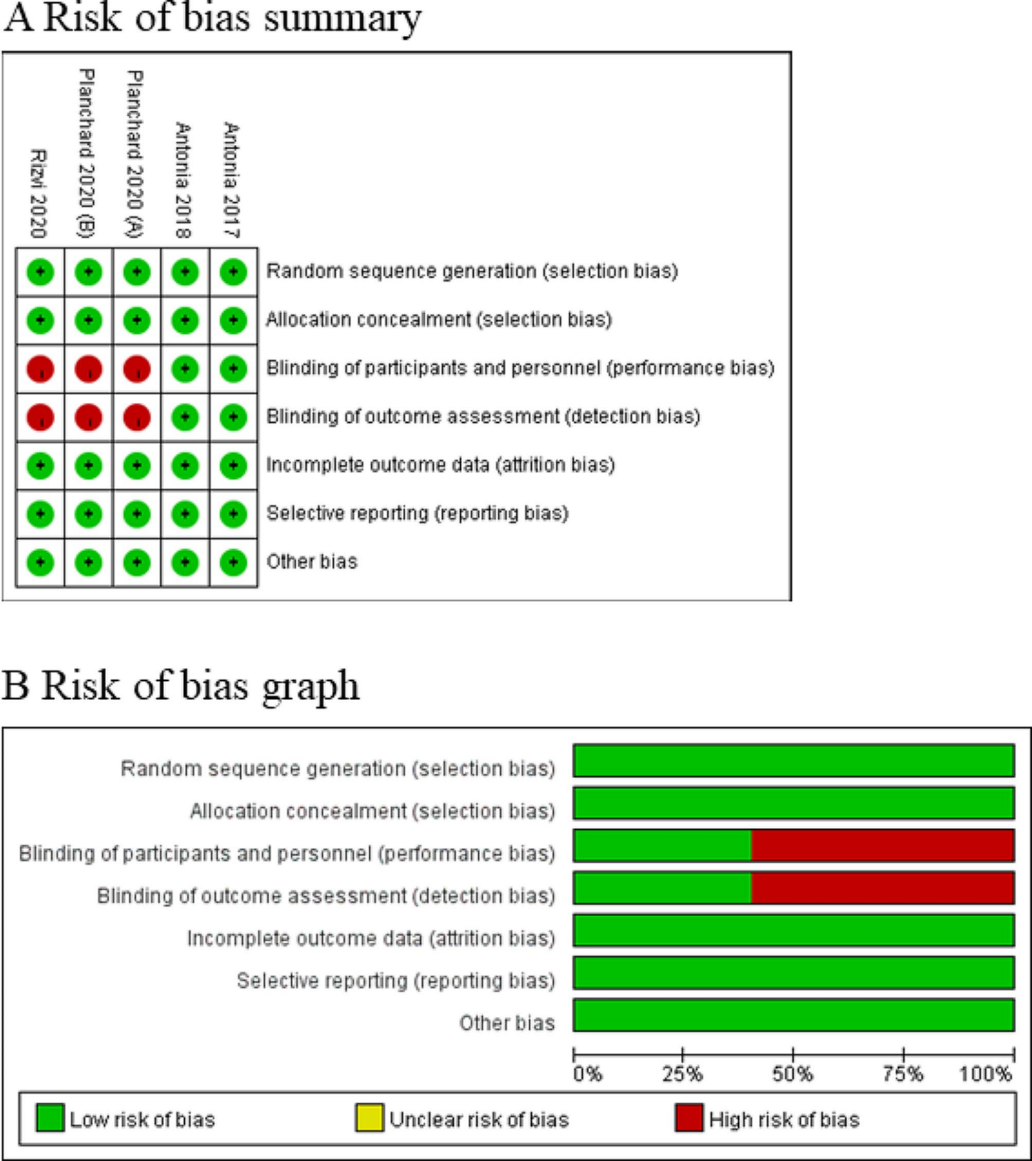
Risk of bias assessment. (**A**) Authors’ judgments about each risk of bias item for each included study. (**B**) Authors’ judgments about each risk of bias item presented as percentages across all included studies

Among the four RCTs, three studies reported survival rate and overall survival [6, 8, 12], three studies reported progression-free survival rate and progression-free survival [6, 12, 18], as well as two studies reported adverse events, grade ≥ 3 adverse events and adverse events [6, 12]. Risk of bias analysis (Fig. 2) showed that two studies had high risk of performance bias and detection bias due to the open label of two groups [6, 12], but all RCTs generally had high quality.

### Primary outcomes: survival rate and overall survival

**Fig. 3:**
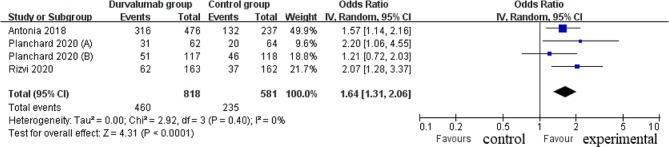
Forest plot for the meta-analysis of survival rate

**Fig. 4:**
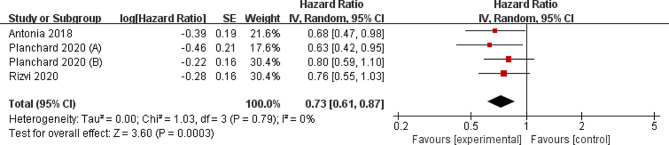
Forest plot for the meta-analysis of overall survival

Compared to control group for NSCLC, durvalumab supplementation was associated with significantly higher survival rate (OR = 1.64; 95% CI = 1.31 to 2.06; *P* < 0.0001) with no heterogeneity among the studies (I^2^ = 0%, heterogeneity *P* = 0.40, Fig. 3) and prolonged overall survival (HR = 0.73; 95% CI = 0.61 to 0.87; *P* = 0.0003) with no heterogeneity among the studies (I^2^ = 0%, heterogeneity *P* = 0.79, Fig. 4).

### Sensitivity analysis

No heterogeneity was observed for the primary outcomes, and thus we did not perform the sensitivity analysis by omitting one study in turn for the meta-analysis.

### Secondary outcomes

**Fig. 5:**
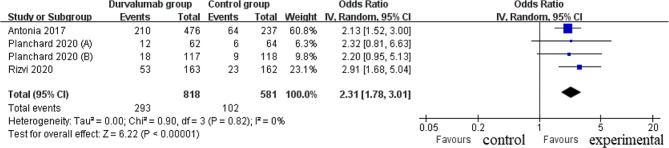
Forest plot for the meta-analysis of progression-free survival rate

**Fig. 6:**
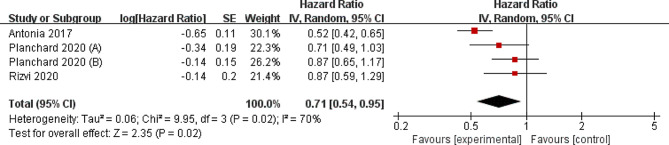
Forest plot for the meta-analysis of progression-free survival

**Fig. 7:**
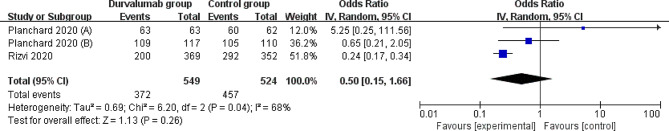
Forest plot for the meta-analysis of adverse events

**Fig. 8:**
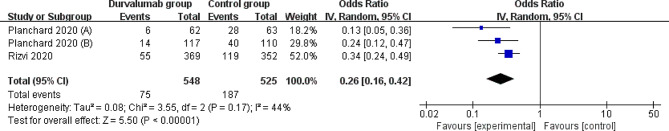
Forest plot for the meta-analysis of grade ≥ 3 adverse events

Compared with control group for NSCLC, durvalumab supplementation showed substantially improved progression-free survival rate (OR = 2.31; 95% CI = 1.78 to 3.01; *P* < 0.00001; Fig. 5) and progression-free survival (HR = 0.71; 95% CI = 0.54 to 0.95; *P* = 0.02; Fig. 6). With regard to the safety, durvalumab supplementation showed no obvious impact on the incidence of adverse events (OR = 0.50; 95% CI = 0.15 to 1.66; *P* = 0.26; Fig. 7), but was associated with substantially reduced grade ≥ 3 adverse events (OR = 0.26; 95% CI = 0.16 to 0.42; *P* < 0.00001; Fig. 8).

## Discussion

In the phase II ATLANTIC trial, durvalumab showed important potential in improving the efficacy for heavily pretreated (third-line or higher) patients with NSCLC when compared with other anti-PD-1/PD-L1 agents [2]. Durvalumab has become an increasingly important immunoglobulin G1 monoclonal antibody to block PD-L1. In order to explore the efficacy of durvalumab supplementation for NSCLC patients, our meta-analysis included four RCTs and 1399 patients. The results suggested that compared to control intervention, durvalumab supplementation substantially benefited to improve survival rate, overall survival, progression-free survival rate and progression-free survival for patients with NSCLC.

In terms of sensitivity analysis, although there was no significant heterogeneity, several factors may produce some bias. Firstly, the stages of NSCLC were different among included RCTs, including metastatic and locally advanced cancers. Squamous and nonsquamous histologic types were both included, and they may have various sensitivity to durvalumab treatment. Thirdly, the treatment duration of durvalumab supplementation varied from 2 years to 3 years, which may affect the efficacy assessment. Indeed, anti-PD-1/PD-L1 therapies have demonstrated clinical benefit in patients with various PD-L1 expression levels, even in those with PD-L1-negative tumors [2, 19]. Additionally, the simultaneous blockade of the PD-1/PD-L1 and cytotoxic T lymphocyte-associated antigen-4 pathways were confirmed to produce additive or synergistic antitumor activity and may be an important treatment option in patients with low/negative PD-L1 expression [20–22].

With regards to the safety, durvalumab supplementation led to similar incidence of adverse events compared to standard chemotherapy, but can remarkably reduce the incidence of grade ≥ 3 adverse events for NSCLC patients. We should also consider several limitations. Firstly, our analysis was based on only four RCTs and more studies with large patient samples should be conducted to confirm this finding. Secondly, the duration of durvalumab treatment were different in the included studies, and may mainly account for some heterogeneity. Thirdly, NSCLC patients with different stages may produce some bias for efficacy assessment.

## Conclusion

Durvalumab supplementation showed improved treatment efficacy for NSCLC patients with reduced incidence of grade ≥ 3 adverse events.

### Electronic supplementary material

Below is the link to the electronic supplementary material.

Supplementary Material 1

## Data Availability

Not applicable.
