# Supplementary material for: Durvalumab supplementation for non-small-cell lung cancer: a meta-analysis study
Source: J Cardiothorac Surg. 2024 Jul 4;19:421. doi: 10.1186/s13019-024-02940-3 (PMC11223428; doi:10.1186/s13019-024-02940-3)
Supplement: Supplementary file 1 — Supplementary Material 1 [file 13019_2024_2940_MOESM1_ESM.docx]

**Supplementary table 1 Searches Run on June, 2022**

| **Databases** | **Search** | **Papers** |
| --- | --- | --- |
| PubMed | “durvalumab” AND “lung cancer” OR “NSCLC” | 122 |
| Embase | “durvalumab” AND “lung cancer” OR “NSCLC” | 58 |
| Web of science | “durvalumab” AND “lung cancer” OR “NSCLC” | 52 |
| EBSCO | “durvalumab” AND “lung cancer” OR “NSCLC” | 48 |
| Cochrane library databases | “durvalumab” AND “lung cancer” OR “NSCLC” | 105 |

**Total papers: 385**
